# Hiccups and Slurring of Speech: Atypical Presentation of High-Altitude Cerebral Edema

**DOI:** 10.7759/cureus.34997

**Published:** 2023-02-14

**Authors:** Nidhi Kaeley, Soumya Subhra Datta, Ankit Sharma, Jithesh G

**Affiliations:** 1 Emergency Medicine, All India Institute of Medical Sciences, Rishikesh, Rishikesh, IND; 2 Internal Medicine, All India Institute of Medical Sciences, Rishikesh, Rishikesh, IND

**Keywords:** cerebral edema, rare finding, slurring of speech, hiccups, high altitude cerebral edema, atypical presentation, hace

## Abstract

High-altitude cerebral edema (HACE) is one of the rare and severe form of high-altitude mountain sickness. Usually it presents as headache, altered mental status, ataxia in un-acclimatized person with rapid ascent to high altitude. Here we report a case of a 62-year-old male patient who had history of rapid ascent to high altitude and presented to the department of emergency after descent from high altitude with an atypical presentation as hiccups and slurring of speech. Magnetic resonance imaging (MRI) of brain showed white matter edema suggestive of HACE. The patient improved after treatment with supplemental oxygen, dexamethasone, and acetazolamide. He was discharged after three days of hospital stay with complete resolution of symptoms.

## Introduction

Acute mountain sickness starts developing at an altitude of 2500m or higher. It has features like headache, dizziness, fatigue, difficult sleeping and other neurological issues [1]. High-altitude cerebral edema (HACE) is a rare and potentially fatal form of acute mountain sickness. HACE usually presents in severe form of high-altitude neurological illness. HACE is described as symptoms of acute mountain sickness with coinciding ataxia or altered mental status. HACE has been reported in unacclimatized persons at an altitude of above 2000m. Though exact incidence of HACE is unclear but is seen rarely [2]. At higher altitudes, hypoxemia leads to cerebral vasodilation which causes capillary leakage and vasogenic cerebral edema occurs. This causes all the neurological presentation of HACE. But neuropsychiatric course and prognosis are still unclear [3]. Here we report a patient who presented with hiccups as only initial symptom followed by slurring of speech.

## Case presentation

A 62-year-old male patient presented to emergency department with history of ascent to Char Dham Yatra in Uttarakhand (India) with his family. After starting from Delhi (300m from sea level) he arrived to Kedarnath Dham (3583m) in two days so his ascent was fairly rapid. Initially on the ascent journey he was in his normal state of good health, but on reaching the destination at 3583m, he started having hiccups. He ignored the symptoms and continued the journey, and then he started having slurring of speech along with nausea and mild headache. With these symptoms he presented to base camp clinic at 1982m, with vitals of blood pressure 160/90 mmHg, heart rate 96/min, respiratory rate 20/min, oxygen saturation 95% room air. He was a known case of hypertension on tablet amlodipine 5 mg for seven years. He was given symptomatic treatment in form of antacids and paracetamol and advised descent.

**Figure 1:**
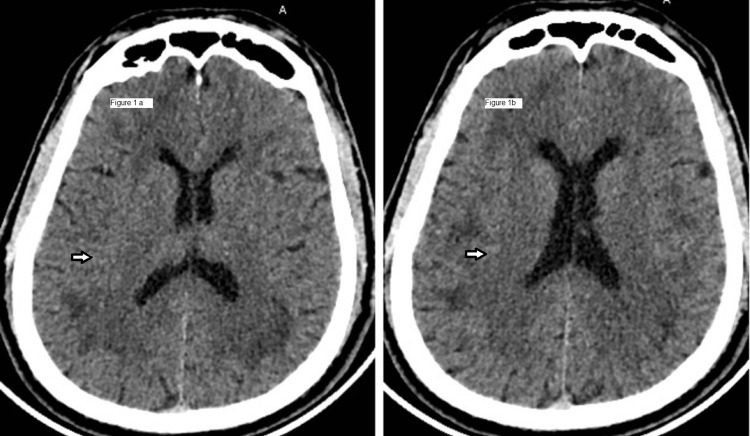
Non-contrast computed tomography (NCCT) head showing bilateral patchy subcortical hypodensities with patchy areas of loss of gray-white matter differentiation in cerebral hemisphere suggestive of white matter edema. Figure 1a and Figure 1b are different cut sections of NCCT head, both showing bilateral patchy hypodensities with patchy areas of loss of gray-white matter differentiation in cerebral hemisphere.

The patient arrived to the emergency department of tertiary care centre in India with complaints of persistent hiccups and slurring of speech. On arrival his blood pressure was 146/98mmHg, respiratory rate 16/min, oxygen saturation 96% room air, pulse rate 80/min, and Glasgow coma scale was 15/15. There was no history of fever, shortness of breath, seizure, head trauma, focal neurological deficit, and decreased urine output (uremic gastritis). His respiratory, cardiac and abdominal systemic examinations were normal. On central nervous system examination, bilateral pupils were normal size and equally reactive to light, power in bilateral upper and lower limb were 5/5, deep tendon reflexes were present and normal, no neck rigidity and Kernig’s, Brudzinski’s signs were negative, cerebellar signs such as nystagmus, dysdiadochokinesia, heel-knee test, finger-nose test were normal, and Romberg sign was negative. Fundus examination showed bilateral mild proliferation. Hematological and biochemical parameters were normal. There was no evidence of hyponatremia or hypoglycemia. The complete blood count, kidney function test and liver function test were all within normal limits. Investigations for Dengue NS-1 antigen, Dengue IgM antibody, Scrub Typhus IgM antibody, Peripheral smear for Malaria were all negative. His chest radiograph showed no signs of pulmonary edema. Non-contrast computed tomography (NCCT) head was done which showed bilateral patchy subcortical hypodensities with patchy areas of loss of gray-white matter differentiation in cerebral hemisphere suggestive of white matter edema (Figure 1a and Figure 1b).

**Figure 2:**
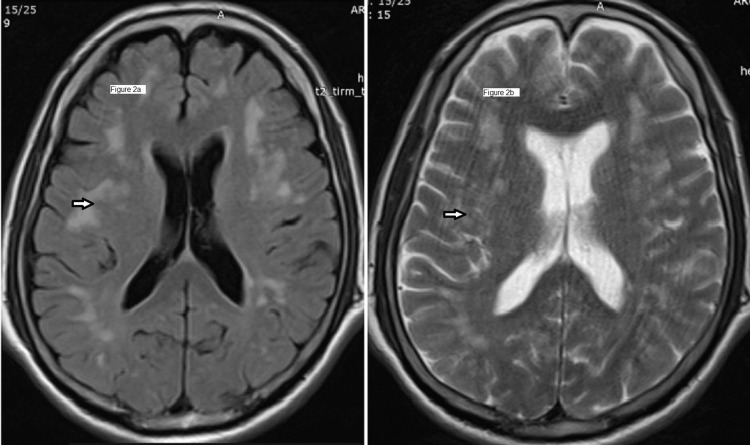
MRI brain showing multiple patchy and confluent areas of hyperintensities in subcortical and periventricular white matter of bilateral cerebral hemispheres in T2-weighted image (T2-WI) and fluid attenuation inversion recovery (FLAIR) image. Figure 2a is MRI brain T2-weighted image (T2-WI) and Figure 2b is MRI brain fluid attenuation inversion recovery (FLAIR) image, both showing patchy and confluent areas of hyperintensities in subcortical and periventricular white matter of bilateral hemispheres.

**Figure 3:**
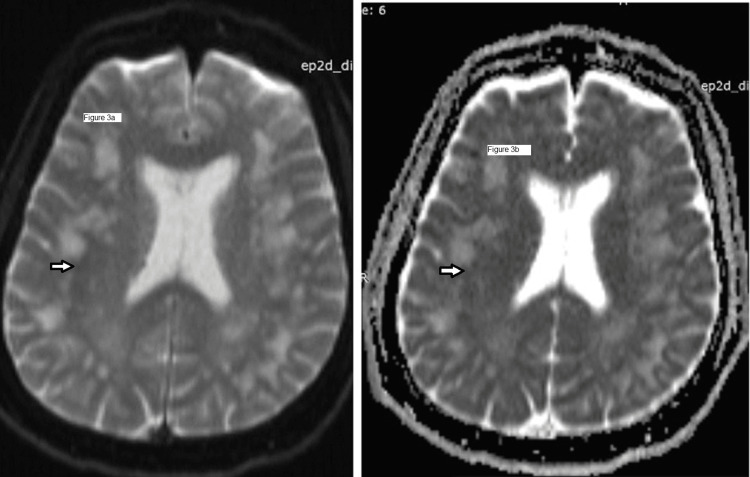
MRI brain diffusion-weighted image (DWI) showing no signal restriction. Figure 3a and Figure 3b are different cut sections of MRI brain diffusion-weighted image (DWI), both showing no signal restriction which is seen in HACE patients on MRI DWI.

Since NCCT head and fundus examination is suggestive of cerebral edema, cerebrospinal fluid (CSF) analysis was not done immediately. Then magnetic resonance imaging (MRI) was done for this patient. The MRI showed multiple patchy and confluent areas of hyperintensities in subcortical and periventricular white matter of bilateral cerebral hemispheres in T2-weighted image (T2-WI) (Figure 2a) and fluid attenuation inversion recovery (FLAIR) image (Figure 2b) which is suggestive of HACE. On MRI brain diffusion-weighted images (DWI) (Figure 3a and Figure 3b), there is no signal restriction which also advocates MRI findings of HACE.

He was given 8mg of intravenous dexamethasone stat followed by 4mg every six hourly and tablet acetazolamide 250mg twice daily orally. He did not require oxygen therapy as he maintained normal oxygen saturation throughout his hospital stay. His hiccups resolved over a night. He was kept under observation in hospital for three days and then discharged in symptom-free state.

## Discussion

HACE is one of the rare and potentially fatal form of acute mountain sickness. HACE is usually seen after two days of rapid ascent to altitude above 4000m but it can also occur at elevations as low as 2500m. At altitudes of 4000-5000m incidence of HACE is 0.5-1%. It is associated with the faster rate of ascent irrespective of gender and age. Prior history of high-altitude illness, abrupt ascent from lower altitude without acclimatization, rapid rate of ascent and heavy physical exertion are the most common risk factors [4].

The patient in our case was hypertensive and Canoui-Poitrine et al. in their cohort study found that hypertension was one of the risk factors in patients developing severe high-altitude illness [5].

Though exact pathophysiology of HACE is not clear, but it is presumed to be the extreme form of acute mountain sickness. At high altitude, neuro-hormonal (Nitric oxide, vascular endothelial growth factor (VEGF), free radicals and cytokines) and hemodynamic responses result in cerebral vasodilatation causing intracranial hypertension leading to capillary leakage causing vasogenic brain edema due to movement of fluid and protein out of vascular compartment. The hypoxia-induced free radical production also causes failure of sodium-potassium ATPase pump resulting in astrocyte swelling from osmotic-oxidative stress causing subsequent cytotoxic edema that contributes to microvascular disruption and microbleeds [4,6].

Patients of HACE usually present with headache, various levels of confusion, abnormal behavior, fatigue, altered consciousness, ataxia and sometimes findings consistent with delirium [3, 7-10]. High altitude headache, a cardinal neurological component of HACE, may be seen in combination with additional symptoms like nausea, vomiting, anorexia, dizziness, fatigue and sleep disturbances [11]. If HACE is not diagnosed and inappropriately treated then coma can develop followed by death from brain herniation within 24 hours [12].

Bolotin et al. reported a case where a patient who ascended to a height of 2829m developed intermittent migraine headache-like symptoms which was later diagnosed as HACE by suggestive findings from MRI study of brain [2]. This case report suggests that HACE can develop at a comparatively lower altitude and may present with atypical symptoms.

Sugimoto et al. stated that a patient with vasogenic edema of medulla oblongata presented with the symptom of hiccups which resolved on resolution of edema when the patient was treated with corticosteroids [13]. Similar findings were reported by Pereira et al. in their case report where a patient with right thalamic lesion with large perilesional vasogenic edema presented with hiccups as one of the presenting symptoms [14]. These reports suggest that vasogenic edema of brain occurring in patients with HACE can also lead to hiccups as a symptom.

Computerized tomography (CT) of brain in HACE patients is usually normal. But with progression of brain edema there is a loss of gray-white matter differentiation which may be detectable in CT scan [15]. MRI brain studies in patients of HACE show reversible edema of predominantly white matter which is evident as hyperintensity on T2-WI and FLAIR images, especially in the subcortical white matter and corpus callosum [16, 17]. The vasogenic edema in HACE does not show any signal restriction in diffusion-weighted MRI brain images [17]. Similar findings were present in the brain imaging of our patient.

Pre-acclimatization and gradual ascent are preventive measures of HACE. Descent and use of supplemental oxygen, dexamethasone, acetazolamide, and hyperbaric oxygen can be used for treatment purpose [18].

In our patient, the presenting features were hiccups followed by slurring of speech. These symptoms are not typical for HACE and have not been reported previously in any study or case report. His brain imaging findings in both CT and MRI were consistent with the findings of reversible vasogenic edema in HACE and after the initiation of treatment with dexamethasone and acetazolamide his symptoms resolved significantly.

## Conclusions

HACE is fatal form of acute mountain sickness. Though exact pathophysiology behind the development of HACE is not very well known, but early diagnosis and immediate intervention is lifesaving. These patients may present with uncommon initial symptoms such as hiccups and slurring of speech. A proper detailed history and a high index of suspicion is required for the early diagnosis and management of these patients. So if there is atypical presentations or unexplained neurological symptoms in any patient with history of high-altitude travel, HACE should be kept as a differential diagnosis.
